# Fast Association Tests for Genes with FAST

**DOI:** 10.1371/journal.pone.0068585

**Published:** 2013-07-23

**Authors:** Pritam Chanda, Hailiang Huang, Dan E. Arking, Joel S. Bader

**Affiliations:** 1 Department of Biomedical Engineering and Institute of Genetic Medicine, Johns Hopkins University, Baltimore, Maryland, United States of America; 2 High Throughput Biology Center, Johns Hopkins University School of Medicine, Baltimore, Maryland, United States of America; 3 Analytic and Translational Genetics Unit, Department of Medicine, Massachusetts General Hospital, Boston, Massachusetts, United States of America; 4 Program in Medical and Population Genetics, Broad Institute of MIT and Harvard, Cambridge, Massachusetts, United States of America; 5 McKusick-Nathans Institute of Genetic Medicine, Johns Hopkins University School of Medicine, Baltimore, Maryland, United States of America; Institut Jacques Monod, France

## Abstract

Gene-based tests of association can increase the power of a genome-wide association study by aggregating multiple independent effects across a gene or locus into a single stronger signal. Recent gene-based tests have distinct approaches to selecting which variants to aggregate within a locus, modeling the effects of linkage disequilibrium, representing fractional allele counts from imputation, and managing permutation tests for p-values. Implementing these tests in a single, efficient framework has great practical value. Fast ASsociation Tests (Fast) addresses this need by implementing leading gene-based association tests together with conventional SNP-based univariate tests and providing a consolidated, easily interpreted report. Fast scales readily to genome-wide SNP data with millions of SNPs and tens of thousands of individuals, provides implementations that are orders of magnitude faster than original literature reports, and provides a unified framework for performing several gene based association tests concurrently and efficiently on the same data. Availability: https://bitbucket.org/baderlab/fast/downloads/FAST.tar.gz, with documentation at https://bitbucket.org/baderlab/fast/wiki/Home

## Introduction

Genome-wide association studies (GWAS) are powerful tools for investigating the genetic basis of common diseases and have revealed new genetic factors for many complex traits [1], [2]. The goal of a GWAS is to establish an association or correlation between a genetic variant and a trait. The tested variants are predominantly single-nucleotide polymorphisms (SNPs), inexpensively genotyped by a variety of platforms. Individual SNP-based tests have a consensus p-value threshold of 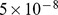 for genome-wide significance.

More recent methods have proposed to test the hypothesis that individual genes can house multiple independent associations and increase power by combining independent associations, whether in protein-coding domains or flanking regulatory regions into a single and stronger aggregated signal. Data sets include SNPs that are genotyped and SNPs computationally imputed from the 1000 Genomes Project [3] or other reference panels [4], [5]. Imputation can improve the power of GWAS to detect disease associated loci [6] and is essential for meta-analysis across platforms that genotype different markers. Imputed data sets are large (∼20 million SNPs using the latest 1000 Genome Project release). As a result, repeated GWAS using different gene-based methods is both CPU and memory intensive.

The following observations motivate our work. First, several gene-based methods have inefficient implementations or are limited to integer allele counts rather than fractional imputed genotypes or genotype dosages common in genome-scale analysis. Second, many methods require similar statistical calculations, making simultaneous calculation of p-values for several methods not much more expensive than running a single method offering opportunities for sharing intermediate results across methods. Shared calculations provide substantial savings in genome-wide analysis because several hundred thousands or more permutation tests are often required to establish gene-based p-values that are significant genome-wide. Shared calculations also permit different methods to be run automatically against the same permutations, eliminating a source of statistical variation in permutation-based tests. Third, highly-cited whole-genome analysis tools such as Plink [7] and Probabel [8] demonstrate the value of providing multiple types of tests, if only for the convenience of simplified driver scripts and unified input/output formats, but to date are limited primarily to single SNP association analysis rather than gene-based tests. No current platforms realize these computational and practical efficiencies for gene-based tests.

We therefore present Fast, a tool for Fast ASsociation Tests for genome-wide SNP data that efficiently integrates and implements several recently proposed test statistics and provides a unified framework for performing several gene based association tests concurrently and genome-wide on the same data set. A brief description of the algorithms and their extensions implemented are given in the next section. The details are presented in the Materials S1. Fast source and executables are available under the GNU Public License from https://bitbucket.org/baderlab/fast/downloads/FAST.tar.gz.

## Methods

### GWiS

This gene-based test uses Bayesian statistics to combine model selection and statistical tests [9]. Let 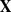 be the genotype matrix with *N* rows (individuals) and *P* columns (SNPs), and 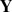 be the 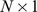 phenotype vector. A model *M* is defined as the subset of *K* SNPs in a gene with *P* total SNPs that are permitted to have non-zero regression coefficients. For each gene, GWiS attempts to find the subset that maximizes the model probability 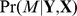. The GWiS test statistic is an approximation to the posterior model probability,
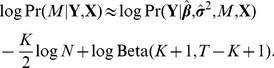
(1)The terms correspond to a standard likelihood ratio test score; a Bayesian Information Criterion (BIC) penalty for replacing full integrals over parameters with maximum likelihood estimates; and a model complexity penalty derived from Bayesian statistics for subset selection. The parameter 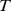 for the subset selection penalty denotes the effective number of tests in a gene calculated from the genotype data independent of phenotypes.

Rather than finding the global optimum, which is NP-hard, GWiS uses a greedy forward search in which the SNP giving the maximal increase to the posterior probability is added to the model sequentially until any remaining SNPs decreases the probability. The forward search uses Gram-Schmidt orthonormalization with sufficient statistic comprising the genotype-genotype covariance matrix, the genotype-phenotype correlations, and phenotype variance, and the marginal allele frequencies and phenotype mean.

As an extension to previous work [9], GWiS now operates directly using this summary data rather than full genotype and phenotype information; this improvement is very important for applications to meta-analysis where only the summary data is available. Furthermore, GWiS now implements Bayesian logistic regression for dichotomous traits using iterative reweighted least squares [10].

### Best SNP in Gene

The MinSNP p-value is the p-value for the best SNP within a gene, computed either directly from a parametric distribution or from permutations for a SNP with low allele frequency. In MinSNP-Gene, single-SNP F-statistics are computed for each SNP within a gene; the best F-statistic within the gene is used as its test statistic; and this is converted to a p-value with gene-based permutations to correct for gene size.

### BIMBAM

Bimbam uses the average Bayes Factor for all possible 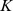-SNP models within a gene as the test statistic. By default the sum is limited to 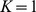, single-SNP models, because computing all 2-SNP models is computationally intensive. The Bayes Factor for a single-SNP model is
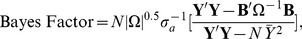
(2)where the number of individuals is 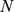; the 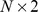 genotype matrix 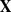 has first column 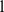 and second column the genotype dosages; 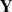 is the phenotype column vector; 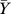 is the scalar phenotype mean; the matrix 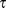 is diagonal with terms 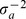, where 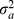 is an adjustable parameter representing the typical additive variance for a SNP; 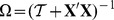; and 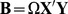 is a 2-component column vector of regression coefficients [11]. We have implemented the test statistic for both genotype dosages and summary data using linear regression for continuous phenotypes and logistic regression for dichotomous phenotypes. The logistic regression model uses the Laplace method to estimate posterior distributions of model parameters, and the distribution modes are obtained using the Fletcher-Reeves conjugate gradient algorithm.

### VEGAS

The Versatile Gene-Based Test for Genome-wide Association [12] uses the sum of single SNP chi-squares as the proposed test statistic for a gene, with p-values corrected for LD. In Fast, the test statistic can be calculated using either linear or logistic models using both genotype dosages and summary data. The significance of each gene is evaluated using permutations when genotype data is available and using simulations for summary data.

### GATES

The Gates test [13] extends the Simes procedure [14] to integrate association evidence from single SNP p-values within a gene. The effective number of independent tests within a gene is denoted 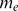 and is estimated from the eigenvalues of the matrix of p-value correlations. With the ascending p-values of 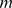 SNPs within a gene denoted 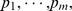 the test statistic is
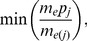
(3)where 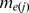 is the effective number of independent p-values among the top 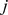 SNPs. The test statistic has an approximate uniform (0,1) distribution and is regarded as the gene's p-value.

### Single SNP

Fast also computes single SNP regression coefficients and standard errors using all input SNPs, whether genic or intergenic, for both linear and logistic regression. This method allows direct comparison of gene-based p-values of the implemented methods with the single SNP p-values and also facilitates discovery of associations from intergenic regions.

### Software features

The application is implemented in C and depends only on the GNU Scientific Library. Command-line options provide access to different methods and parameters. Single chromosomes or regions can be specified, permitting easy parallelization of different regions across multiple compute nodes. When multiple CPUs are available in a node, Fast further allows multi-threaded parallelization of permutations. To reduce memory footprint, Fast processes each chromosome gene-by-gene and retains only the SNPs mapped to that particular gene. Covariates can be specified along with genotype data and are included in all models explored. In absence of genotype data, when the phenotype is case-control, Fast uses linear regression to approximate the calculations of the test statistics for GWiS and Bimbam (See Materials S2). Fast can run several different tests simultaneously and a script (with dependencies on Perl) combines the results into a single output file.

#### Computation of genotype-genotype inner products from reference populations

Several methods require genotype-genotype and genotype-phenotype inner products, which must be estimated from reference populations such as 1000 Genomes when only summary data (single SNP regression coefficients and standard errors, phenotype mean and variance) are available. The covariance 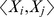 for SNPs 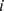 and 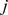 is estimated from covariances 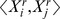 in the reference population as
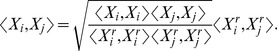
The variance for SNP 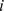 with minor allele frequency 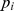 is
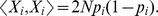
The genotype-phenotype inner products are computed using the summary data SNP regression coefficients 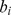 and standard errors 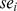:
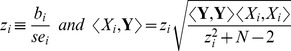
Pre-computing the inner products (as implemented in Vegas) results in SNP-SNP correlation matrices that are extremely high dimensional and occupy several gigabytes. We have instead compute the inner products for a given pair of SNPs dynamically when needed. SNP data is read using pre-generated index files for memory- and CPU-efficient random file access to haplotype data. Pre-computed haplotype files and their corresponding index files from the 1000 Genomes project (release May 2012) are available from https://bitbucket.org/baderlab/fast/wiki/RefHaps.

### Permutations

For each implemented test statistic except Gates, p-values are obtained using permutations. When individual level genotype and phenotype data are available, permutations are conducted at the gene level using the Fisher-Yates shuffle algorithm [15].

For summary data, the empirical p-values are computed by simulating z-scores under the null using random variates sampled from a multivariate normal distribution with covariance matrix computed from the linkage disequilibrium between the SNPs in an appropriate reference population. Under the null, for large N, the z-score for a single SNP is approximately Normal(0,1). If 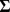 denotes the correlation matrix for the 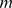 SNPs in a gene, under the null, the correlation matrix among the z-scores is also 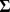. Therefore, the null distribution of the z-scores in a gene is multivariate normal with mean 0 and correlation matrix 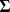. Permutations are performed by simulating this distribution using LDL factorization of the correlation matrix: 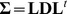 , in which 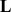 is unit lower triangular and the matrix 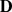 is diagonal. Details are discussed in Materials S1.

## Results

To evaluate the performance of Fast, case-control data containing 500 cases and 5500 controls with genotypes from 10,000 independent SNPs were simulated using Plink [7]. Out of the 10,000 SNPs, 10 SNPs were simulated to be disease associated with a multiplicative risk of 1.5 for the homozygotes. In addition, 182 genes were simulated covering all the SNPs sequentially without overlap, starting from the base-pair position of the first SNP. Each gene has length uniformly random between 10 and 100 SNPs, with an average of 55 SNPs per gene. To obtain runtime performance of each implemented test, each test statistic was computed independently and p-values were obtained with 1000 permutations using genotype data, or with 1 million permutations using summary data from single SNP analysis (Table 1, AMD Opteron 2.3 GHz CPU, 7.8 GB RAM). Also, available standalone implementations of Bimbam version 1.0 (http://www.bcm.edu/cnrc/mcmcmc/bimbam) and Plink were run with genotype data with both linear and logistic models; Vegas version 0.8.27 (http://gump.qimr.edu.au/VEGAS/) and Gates version 2.0 (http://bioinfo1.hku.hk:13080/kggweb/) were run with summary data from single SNP analysis. Fast has smaller or substantially shorter runtime, with similar or substantially reduced memory requirements (Table 1).

**Table 1:** Runtime and memory usage in Fast using simulated data compared with publicly available stand-alone implementations (denoted Orig).

		Runtime			Max memory usage		
Method	Implementation	Linear	Logistic	Summary	Linear	Logistic	Summary
Gwis	Fast	3.61	33.72	4.85	120	112	290
Bimbam	Fast	4.00	102.85	5.27	110	111	280
Bimbam	Orig	392	>7200	-	125	≥125	-
Vegas	Fast	5.34	89.40	8.00	110	112	286
Vegas	Orig	-	-	14.7	-	-	3030
Minsnp	Fast	4.70	64.80	3.10	110	124	282
Minsnp-gene	Fast	5.58	113.40	6.90	110	112	275
Gates *	Fast	2.90	4.00	0.37	103	103	270
Gates *	Orig	-	-	0.87	-	-	230
Single-SNP*	Fast	0.28	1.50	-	80	80	-
Single-SNP*	Plink	1.83	2.12	-	140	140	-
All	Fast	4.85	186.04	11.00	122	128	300

All runtimes are in minutes and memory usages are in megabytes. ‘Linear/Logistic’ uses genotype data while ‘Summary’ uses summary data. All indicates running Gwis, Bimbam, Vegas, Minsnp, Minsnp-gene and Single-snp simultaneously in Fast.

* No permutations.

## Discussion

Fast provides an integrated whole-genome analysis platform for several gene-based tests as well as conventional single SNP tests. While single-SNP GWAS have been quite successful in identifying many genetic associations for human diseases [16], [17], they can miss true associations arising from multiple independent associations within a single gene. Gene-based tests complement the traditional univariate GWAS, can improve power when single-SNP tests did not reach genome-wide significance, can identify how many independent effects are within a genomic region, and are directly compatible with gene-based analysis of networks and pathways.

By combining several gene-based tests in a single application, Fast provides several advantages:

- Despite the evidence that gene-based tests can be more powerful than single SNP based tests, different gene-based tests are likely to perform better under different genetic architectures (multiple independent signals vs. single signal in a gene, size of gene, patterns of LD in the gene). Therefore, Fast provides a natural platform to run several gene-based tests concurrently and enable identification of significant associations using the best performing test.
- Fast improves the basic capabilities of the existing gene-based tests. Gwis is extended to use logistic model for dichotomous traits; both Gwis and Bimbam are improved to run with summary data; and all methods are enabled for real-valued imputed data rather than integer allele counts.
- The implementations of the existing tests are substantially improved, reducing CPU and memory requirements. When multiple CPUs are available, Fast further boosts performance by parallelizing permutations.
- Efficient computation of multiple tests simultaneously is achieved by taking advantage of shared calculations and data structures.
- Fast eliminates the nuisance of separate data formats and driver scripts for each method, and provides an additional control by running each method on the same set of permutations.
- Fast integrates the output from different tests into a single file for cross-comparison.

The test statistics incorporated in Fast have discovered several gene-based associations missed by single-SNP tests: the PPRAD gene for fasting insulin [18]; genes CYP2C9 and ADORA2A for caffeine intake [19]; clusters of genome-wide significant markers located using gene-based tests at chromosomes 19p12, 11q25, and 8p23.2 [20]; gene locus SCN5A-SCN10A for ECG QRS interval [9]. While other gene-based tests have been recently proposed [21], [22], the tests currently implemented in Fast are chosen to provide a mix of frequentist and Bayesian motivated test statistics. The newer tests will be incorporated into our application in future releases depending on their usage in gene-based association studies.

Fast provides gene-based statistics for common variants that can potentially be combined with gene-based tests for rare variants discovered by exome or whole-genome sequencing. Whole-genome rare variant analysis methods are still being developed, with no clear consensus on the best methods. When the dominant methods become clear, Fast will be an ideal platform to extend to rare-variant tests.

With the rapidly increasing count of SNPs available for GWAS and availability of imputed genotypes, our application reduces the time and cost of running several gene-based analysis methods as well as single SNP tests genome-wide and facilitates discovery of potentially unknown disease causing genes through comparison and assimilation of output from the different tests.

## Supporting Information

Materials S1 FAST.
(PDF)

Materials S2 **Figure S1, Estimated power of the methods to detect the simulated gene under linear and logistic regression models. Figure S2, Comparing single SNP chi-squares and p-values between linear and logistic regression with genotype data for N = 1000, 3000 and 5000. Figure S3, Comparing minSNP Gene test statistic and gene p-values between logistic regression (genotype data) and linear regression (summary data) and for N = 1000, 3000 and 5000. Figure S4, Comparing Vegas test statistic and gene p-values between logistic regression (genotype data) and linear regression (summary data) for N = 1000, 3000 and 5000. Figure S5, Comparing Bimbam test statistic and gene p-values between logistic regression (genotype data) and linear regression (summary data) for N = 1000, 3000 and 5000. Figure S6, Comparing GWiS test statistic and gene p-values between logistic regression (genotype data) and linear regression (summary data) for N = 1000, 3000 and 5000.** Only models with test statistic >0 undergo permutations to generate P-values. **Figure S7, Comparing Gates p-values between logistic regression (genotype data) and linear regression (summary data) for N = 1000, 3000 and 5000. Figure S8, Comparing minSNP test statistic and gene p-values between logistic regression (genotype data) and linear regression (summary data) for N = 1000, 3000 and 5000.**
(PDF)
