# Supplementary material for: Fast Association Tests for Genes with FAST
Source: PLoS One. 2013 Jul 23;8(7):e68585. doi: 10.1371/journal.pone.0068585 (PMC3720833; doi:10.1371/journal.pone.0068585)
Supplement: Materials S1 — FAST. (PDF) [file pone.0068585.s001.pdf]

FAST : Supplementary 1

Pritam Chanda, Hailiang Huang, Dan E. Arking, Joel S. Bader

Contents

1 Bayesian model selection with GWiS **1**

1.1 Overview 1

1.2 Computing the effective number of tests 4

1.3 Fast Model Search with Gram-Schmidt orthonormalization 4

1.4 GWiS using summary data 5

1.5 GWiS for dichotomous traits using iterative reweighted least squares 6

2 BimBam and its extensions **7**

2.1 Computation of Bayes Factor using linear regression model 8

2.2 Computation of Bayes Factor using logistic regression 9

3 Simulations in the Absence of Genotype Data **10**

4 Creating a valid correlation matrix **11**

1 Bayesian model selection with GWiS

1.1 Overview

The GWiS score has three components: a model complexity penalty derived from Bayesian statistics for subset selection; a Bayesian Information Criterion (BIC) penalty for replacing full integrals over parameters with maximum likelihood estimates; and a standard likelihood ratio test score. GWiS uses a greedy forward search in which the SNP giving the maximal increase to the model likelihood is added to the model sequentially until all remaining SNPs result in decrease in model likelihood. The phenotype vector $\mathbf{Y}$ for N individuals is an $N \times 1$ vector of trait values. The genotype matrix $\mathbf{X}$ has N rows and P columns, one for each of P genotyped markers assumed to be biallelic SNPs.

A model M is defined as the subset of K SNPs in a gene with P total SNPs that are permitted to

have non-zero regression coefficients. For each gene, GWiS attempts to find the subset that maximizes the model probability $\Pr(M|\mathbf{Y},\mathbf{X})$, where each of the P columns of $\mathbf{X}$ corresponds to a SNP assigned to the gene. According to Baye's rule,

$$\Pr(M|\mathbf{Y},\mathbf{X}) = \Pr(\mathbf{Y}|M,\mathbf{X})\Pr(M)/\Pr(\mathbf{Y}|\mathbf{X}). \tag{1}$$

The factor $\Pr(\mathbf{Y}|\mathbf{X})$ is model-independent and can be ignored.

The prior probability of the model, $\Pr(M)$, assumes that each of the P SNPs within the gene has an identical probability of being associated with the trait. This probability, denoted f, is unknown, and is integrated out with a uniform prior. The prior is also designed to make the model probability insensitive to SNP density: it should be unaffected if an existing SNP is replicated to create a new SNP marker with identical genotypes. We do this by replacing the number of SNPs within a gene with an effective number of tests, T, calculated from the local LD within a gene. Correlations between SNPs make the effective number of tests smaller than the number of SNPs. With no LD, $T = P$, and with complete LD, $T = 1$. Thus, the model prior based on the effective number of tests is

$$\Pr(M) = \int_0^1 f^K(1-f)^{T-K}df \equiv \mathrm{Beta}(K+1, T-K+1), \tag{2}$$

or $K!(T-K)!/(T+1)!$ for integer values. As the effective number of tests, T, whose calculation is described below, is generally non-integer, we use the standard Beta function rather than factorials.

From Eq. 1, the expression $\Pr(\mathbf{Y}|M,\mathbf{X})$ is again evaluated using Baye's rule. Let $\boldsymbol{\theta}$ denote the parameter vector for model M. Then the posterior probability of the model parameters is

$$\begin{aligned}\Pr(\boldsymbol{\theta}|M,\mathbf{Y},\mathbf{X}) &= \frac{\Pr(\mathbf{Y}|\boldsymbol{\theta},M,\mathbf{X})\Pr(\boldsymbol{\theta})}{\Pr(\mathbf{Y}|M,\mathbf{X})} \\ &= \frac{\Pr(\mathbf{Y}|\boldsymbol{\theta},M,\mathbf{X})\Pr(\boldsymbol{\theta})}{\int_{\boldsymbol{\theta}}\Pr(\mathbf{Y}|\boldsymbol{\theta},M,\mathbf{X})\Pr(\boldsymbol{\theta})d\boldsymbol{\theta}}\end{aligned} \tag{3}$$

Let $f(\boldsymbol{\theta}) = Pr(\mathbf{Y}|\boldsymbol{\theta},M,\mathbf{X})\Pr(\boldsymbol{\theta})$. If $\hat{\boldsymbol{\theta}}$ is the maximum likelihood estimate of $\boldsymbol{\theta}$, the Laplace approximation for $\log f(\boldsymbol{\theta})$ is

$$\begin{aligned}\log f(\boldsymbol{\theta}) &\approx \log f(\hat{\boldsymbol{\theta}}) - (1/2)(\boldsymbol{\theta}-\hat{\boldsymbol{\theta}})^T\boldsymbol{\Sigma}(\boldsymbol{\theta}-\hat{\boldsymbol{\theta}}) \\ \text{or, } f(\boldsymbol{\theta}) &\approx f(\hat{\boldsymbol{\theta}})\exp[-(1/2)(\boldsymbol{\theta}-\hat{\boldsymbol{\theta}})^T\boldsymbol{\Sigma}(\boldsymbol{\theta}-\hat{\boldsymbol{\theta}})]\end{aligned} \tag{4}$$

where $\mathbf{\Sigma}$ is given by $-\nabla\nabla \log f(\boldsymbol{\theta})|_{\boldsymbol{\theta}=\hat{\boldsymbol{\theta}}}$.

Using Eq. 4, the denominator of 3 is

$$\begin{aligned}
\Pr(\mathbf{Y}|M,\mathbf{X}) &= \int_{\boldsymbol{\theta}} f(\boldsymbol{\theta})d\boldsymbol{\theta} \\
&= f(\hat{\boldsymbol{\theta}}) \int_{\boldsymbol{\theta}} \exp[-(1/2)(\boldsymbol{\theta}-\hat{\boldsymbol{\theta}})^T \mathbf{\Sigma}(\boldsymbol{\theta}-\hat{\boldsymbol{\theta}})]d\boldsymbol{\theta} \\
&= f(\hat{\boldsymbol{\theta}}) \frac{(2\pi)^{K/2}}{|\mathbf{\Sigma}|^{1/2}} \\
\text{or, } \log \Pr(\mathbf{Y}|M,\mathbf{X}) &= \log \Pr(\mathbf{Y}|\hat{\boldsymbol{\theta}},M,\mathbf{X}) + \log \Pr(\hat{\boldsymbol{\theta}}) + \frac{K}{2}\log 2\pi - \frac{K}{2}\log|\mathbf{\Sigma}|. \qquad (5)
\end{aligned}$$

Assuming that the Gaussian prior distribution over $\boldsymbol{\theta}$ is broad, and $\mathbf{\Sigma}$ is full rank, we approximate Eq. 5 using the steepest descents approximation as in the Schwarzian Bayesin Information Criterion (BIC),

$$\log \Pr(\mathbf{Y}|M,\mathbf{X}) \approx \log \Pr(\mathbf{Y}|\hat{\boldsymbol{\theta}},M,\mathbf{X}) - \frac{K}{2}\log N. \qquad (6)$$

With these approximations, Eq. 1 becomes

$$\log \Pr(M|\mathbf{Y},\mathbf{X}) \approx \log \Pr(\mathbf{Y}|\hat{\boldsymbol{\theta}},M,\mathbf{X}) - \frac{K}{2}\log N + \log \mathrm{Beta}(K+1, T-K+1) \qquad (7)$$

This is the objective function GWiS seeks to maximize using a greedy model search in which the SNP giving the maximum increase in the objective function is added to the model.

Using Eq. 7, we model the phenotype vector $\mathbf{Y}$ using both linear regression (GWiS-Lin) and logistic regression (GWiS-Log) when the $\mathbf{Y}$ is binary.

A standard regression model estimates the phenotype vector as $\mathbf{Y} = \mathbf{X}\mathbf{b} + \mathbf{e}$, where $\mathbf{b}$ is a vector of regression coefficients and $\mathbf{e}$ is a vector of residuals assumed to be independent and normally distributed with mean 0 and variance σ^2 . The log probability of the phenotypes given these parameters is

$$\log \Pr(\mathbf{Y}|\mathbf{b},\sigma^2,\mathbf{X}) = -\frac{1}{2}\left\{N\log(2\pi) + N\log(\sigma^2) + \frac{|\mathbf{Y}-\mathbf{X}\mathbf{b}|^2}{\sigma^2}\right\}. \qquad (8)$$

The maximum likelihood estimators (MLEs) are $\hat{\sigma}^2 = |\mathbf{Y}-\mathbf{X}\mathbf{b}|^2/N$ and $\hat{\mathbf{b}} = (\mathbf{X}'\mathbf{X})^{-1}\mathbf{X}'\mathbf{Y}$, where $\mathbf{X}'$ denotes the transpose $\mathbf{X}$. The total sum-of-squares (SST) is $|\mathbf{Y}|^2$, and the sum-of-squares of the model (SSM) is $|\hat{\mathbf{Y}}|^2 = \mathbf{Y}'\mathbf{X}(\mathbf{X}'\mathbf{X})^{-1}\mathbf{X}'\mathbf{Y}$. The sum-of-squares of the errors or residuals (SSE) is SST $-$

SSM $=|\mathbf{Y}-\mathbf{X}\hat{\mathbf{b}}|^2 = |\mathbf{Y}|^2 - |\hat{\mathbf{Y}}|^2$.

1.2 Computing the effective number of tests

The method we adopt is based on multiple linear regression of SNPs on SNPs. The genotype vector $\mathbf{x}_i$ for each SNP i is standardized to have zero mean. Correlations between all pairs of SNPs i and j are initialized as $C_{ij} = \mathbf{x}_i'\mathbf{x}_j/\sqrt{|\mathbf{x}_i||\mathbf{x}_j|}$. Each SNPs weight w_i is initialized to 1, and the number of effective tests T is initialized to 0. The SNP i with maximum weight is identified, and the following updates are executed:

$$\begin{aligned} T &\leftarrow T + w_i \\ w_j &\leftarrow \max(w_j - C_{ji}^2 w_i, 0) \text{ for all SNPs } j. \end{aligned} \tag{9}$$

This process continues until all weights are equal to zero. When SNPs with maximum weight are tied (as occurs for the first SNP processed), the SNP with lowest genomic coordinate is selected to ensure reproducibility; we have ensured that this method is robust to other methods for breaking ties, including random selection. For simplicity, the correlations are not updated (the update rule would be $C_{jk} \leftarrow \max[C_{jk} - C_{ji}C_{ki}/w_i, 0]$), which may lead to an overestimate for T. Model selection may therefore have a conservative bias. The p-values are not affected, however, because they are calculated by permutation tests as described below.

1.3 Fast Model Search with Gram-Schmidt orthonormalization

With linear regression the model search is implemented efficiently using a Gram-Schmidt orthonormalization process. The greedy model search estimates the phenotype vector $\mathbf{Y}$ by the linear model $\mathbf{Y} = \mathbf{X}\mathbf{b} + \mathbf{e}$. For a model containing P SNPs, the genotype matrix $\mathbf{X}$ has N rows and P columns, one for each of P genotyped markers assumed to be biallelic SNPs. Let's denote the kth column (SNP) of $\mathbf{X}$ by X_k. The SNPs are augmented by X_0, the column vector $\mathbf{1}$, whose regression coefficient is the mean of $\mathbf{Y}$. Using Gram-Schmidt procedure, we can transform the columns of $\mathbf{X}$ into a set of orthonormal vectors Z_k for $k = 1...P$,

$$Z_k = X_k - \sum_{j=0}^{k-1} \mathbf{Proj}(X_k, Z_j) \tag{10}$$

where $\mathbf{Proj}(X_k, Z_j) = \frac{\langle X_k, Z_j \rangle}{\langle Z_j, Z_j \rangle} Z_j$ defines the projection of the vector X_k onto Z_j, $\langle ., . \rangle$ denoting the inner product operation. Here $Z_0 = X_0$. The sum-of-squares of the errors (SSE) of the model $\mathbf{Y} = X_0 + X_1 + \cdots + X_P + \mathbf{e}$ is then same as that of $\mathbf{Y} = Z_0 + Z_1 + \cdots + Z_P + \mathbf{e}$ and is given by $\langle \mathbf{Y} - \hat{\mathbf{Y}}, \mathbf{Y} - \hat{\mathbf{Y}} \rangle$. As the vectors Z_k are orthogonal, $\mathbf{Y}$ can be regressed on each Z_k separately to get the phenotype projections $\hat{Y_k} = \frac{\langle Z_k, \mathbf{Y} \rangle}{\langle Z_k, Z_k \rangle} Z_k$. Then $\hat{\mathbf{Y}}$ can be expressed as $\sum_{k=0}^{P} \hat{Y_k}$. Then the SSE of the P-SNP model SSE_P is

$$\begin{aligned}
\text{SSE}_P &= \langle \mathbf{Y} - \hat{\mathbf{Y}}, \mathbf{Y} - \hat{\mathbf{Y}} \rangle \\
&= \langle \mathbf{Y}, \mathbf{Y} \rangle - 2\langle \mathbf{Y}, \sum_{k=0}^{P} \hat{P_k} \rangle + \langle \sum_{k=0}^{P} \hat{P_k}, \sum_{k=0}^{P} \hat{P_k} \rangle \\
&= \langle \mathbf{Y}, \mathbf{Y} \rangle - 2 \sum_{k=0}^{P} \frac{\langle \mathbf{Y}, Z_k \rangle^2}{\langle Z_k, Z_k \rangle} + \sum_{k=0}^{P} \frac{\langle \mathbf{Y}, Z_k \rangle^2}{\langle Z_k, Z_k \rangle} \\
&= \langle \mathbf{Y} - \overline{\mathbf{Y}}, \mathbf{Y} - \overline{\mathbf{Y}} \rangle - \sum_{k=1}^{P} \frac{\langle \mathbf{Y}, Z_k \rangle^2}{\langle Z_k, Z_k \rangle} \qquad (11)
\end{aligned}$$

The expression $\frac{\langle \mathbf{Y}, Z_k \rangle^2}{\langle Z_k, Z_k \rangle}$ is given by $\langle \mathbf{Y}, X_k \rangle - \sum_{i=0}^{k-1} \frac{\langle \mathbf{Y}, Z_i \rangle \langle X_k, Z_i \rangle}{\langle Z_i, Z_i \rangle}$ where $\langle X_k, Z_i \rangle = \langle X_k, X_i \rangle - \sum_{t=0}^{i-1} \frac{\langle X_k, Z_t \rangle \langle X_i, Z_t \rangle}{\langle Z_t, Z_t \rangle}$ and $\langle Z_i, Z_i \rangle = \langle X_i, X_i \rangle - \sum_{t=0}^{i-1} \frac{\langle X_i, Z_t \rangle}{\langle Z_t, Z_t \rangle}$. Clearly, only the genotype-genotype inner products $\langle X_i, X_j \rangle$, phenotype-phenotype inner product $\langle \mathbf{Y} - \overline{\mathbf{Y}}, \mathbf{Y} - \overline{\mathbf{Y}} \rangle$ and genotype-phenotype inner products $\langle \mathbf{Y}, X_i \rangle$ are sufficient to compute the SSE. Also, only the SSE is sufficient to compute the GWiS objective function (Eq. 7) as the data likelihood depends only on SSE (Eq. 8). Consequently, the model search is very efficient as all the inner product computations in an iteration can be used in future iterations.

1.4 GWiS using summary data

To calculate the GWiS test statistic using GWiS-Lin, it is necessary to calculate the sum-of-squares of the errors (SSE) of the model, which is the difference between total sum-of-square (SST) and sum-of-square of the model (SSM). SST is available in the summary data as phenotype variance × the number of individuals. If the SNPs are independent from one another, SSM of a model with k SNPs is the summation of the SSMs from each of the k SNPs. In reality, SNPs are correlated and it is necessary to properly adjust the SSMs of the SNPs in the model for the correlations among them. Gram-Schmidt Orthonormalization provides an iterative procedure to adjust for the correlations among SNPs in the summation.

From the above equations we can see that GWiS-Lin only requires the genotype-genotype inner

products $\langle X_i, X_j \rangle$, phenotype-phenotype inner product $\langle \mathbf{Y} - \overline{\mathbf{Y}}, \mathbf{Y} - \overline{\mathbf{Y}} \rangle$ and genotype-phenotype inner products $\langle \mathbf{Y}, X_i \rangle$ to compute the GWiS objective function and do the model search. These inner products can be easily computed from the covariances between the SNPs and the trait, $\mathrm{COV}(\mathbf{Y}, \mathrm{X_i})$, and the covariance matrix from the SNPs, $\mathrm{COV}(\mathrm{X_i}, \mathrm{X_j})$, because inner product between two vectors A and B of length N is $\langle A, B \rangle = (N-1)\mathrm{COV}(A, B) + N(\overline{A})(\overline{B})$. Recall that $\mathbf{Y}$ is the phenotype vector and X_i, X_j is the genotype vector of SNP i and j (i-th and j-th column of the genotype matrix $\mathbf{X}$). For each iteration, SNP that has the best SSM is selected (denoted as $\mathrm{SSM_{best}}$). We then add its SSM ($\mathrm{SSM_{best}}$) to the total SSM ($\mathrm{SSM} = \mathrm{SSM} + \mathrm{SSM_{best}}$) and adjust the SSMs of the remaining SNPs through the updates

$$\begin{aligned} \mathrm{COV}(\mathbf{Y}, X_i) &\leftarrow \mathrm{COV}(\mathbf{Y}, X_i) - \mathrm{COV}(X_m, X_i)\frac{\mathrm{COV}(\mathbf{Y}, X_m)}{\mathrm{COV}(X_m, X_m)} \\ \mathrm{COV}(X_i, X_j) &\leftarrow \mathrm{COV}(X_i, X_j) - \mathrm{COV}(X_{best}, X_i)\frac{\mathrm{COV}(X_j, X_{best})}{\mathrm{COV}(X_{best}, X_{best})} \\ \mathrm{SSM}_i &= \frac{\mathrm{COV}(\mathbf{Y}, X_i)^2}{\mathrm{COV}(X_i, X_i)}, \end{aligned} \tag{12}$$

in which $\mathrm{COV}(A, B)$ is the covariance of vectors A and B.

To initialize the calculation, Eq. 12 requires the covariances between the SNPs and the trait, $\mathrm{COV}(\mathbf{Y}, X_i)$, and the covariance matrix of the SNPs, $\mathrm{COV}(X_i, X_j)$. The SNP-trait covariances can be calculated from z-scores (usually reported), or can be calculated from the summary information. The z-scores and $\mathrm{COV}(\mathbf{Y}, X_i)$ are related through

$$z_i \equiv \beta_i/\mathrm{SE}_i = \frac{\mathrm{COV}(\mathbf{Y}, X_i)\sqrt{N-2}}{\sqrt{\mathrm{COV}(\mathbf{Y}, Y)\mathrm{COV}(X_i, X_i) - \mathrm{COV}(\mathbf{Y}, X_i)^2}}. \tag{13}$$

The variance of the trait, $\mathrm{COV}(\mathbf{Y}, \mathbf{Y})$, is provided by the summary data, and the variance of the genotype, $\mathrm{COV}(X_i, X_i)$, may be estimated from the mean allele frequency assuming Hardy-Weinberg Equilibrium. The covariance matrix of the SNPs can be estimated from a proxy population, such as Hapmap [1] or 1000 Genomes [2].

1.5 GWiS for dichotomous traits using iterative reweighted least squares

With binary logistic regression, the model search procedure is similar to linear regression wherein the maximum likelihood estimate of the data log likelihood is estimated using an iterative reweighed least squares algorithm based on the Newton-Raphson [3] algorithm to solve the score equations of logistic

regression. For a data set containing P SNPs, the genotype matrix $\mathbf{X}$ has N rows and P columns. The phenotype vector is assumed to be binary containing values $\{1, -1\}$. The data log likelihood is

$$L(\mathbf{b}) = \log \Pr(\mathbf{Y}|\mathbf{b}, \mathbf{X}) = -\sum_{i=1}^{N} \log[1 + \exp(-Y_i {X_i}^T \mathbf{b})], \tag{14}$$

where ${X_i}^T$ denotes the ith row (or sample) of $\mathbf{X}$. To compute the maximum likelihood estimate of $L(\mathbf{b})$, the gradient $\mathbf{g}$ of the data log likelihood is

$$\mathbf{g} = \nabla_{\mathbf{b}} L(\mathbf{b}) = \sum_{i=1}^{N} (1 + \exp(Y_i {X_i}^T \mathbf{b})^{-1}, \tag{15}$$

and the Hessian $\mathbf{h}$ is

$$\mathbf{h} = \nabla\nabla_{\mathbf{b}} L(\mathbf{b}) = -\mathbf{X}^T \mathbf{Q} \mathbf{X}. \tag{16}$$

The diagonal matrix $\mathbf{Q}$ has entries $q_{ii} = [1 + \exp(-Y_i {X_i}^T \mathbf{b})]^{-1} [1 + \exp(Y_i {X_i}^T \mathbf{b})]^{-1}$. At each step of the iterative algorithm, the regression coefficients $\mathbf{b_{new}}$ are recalculated as

$$\mathbf{b_{new}} = \mathbf{b_{old}} - \mathbf{h^{-1} g} \tag{17}$$

Iterations terminate when the difference between the regression coefficient vector $\mathbf{b}$ in two successive iterations is $\leq 10^{-18}$. The resulting coefficient vector $\hat{\mathbf{b}}$ is used to compute the maximum likelihood estimation of the log likelihood $\log \Pr(\mathbf{Y}|\hat{\mathbf{b}}, \mathbf{X})$.

2 BimBam and its extensions

This method uses the average Bayes Factor (BF) for all possible K-SNP models within a gene as the test statistic. For $K \geq 1$, because testing all possible K-SNP models is computationally infeasible and 1-SNP models were found to have as much power as 2-SNP models, Bimbam by default restricts its sum to all 1-SNP models within a gene.

2.1 Computation of Bayes Factor using linear regression model

For a single SNP i, let $\mathbf{X}$ be the $N \times 2$ genotype matrix, with the first column being the column vector $\mathbf{1}$ and the second column being the genotype dosages, i.e,

$$\mathbf{X} = \begin{bmatrix} 1 & X_{i1} \\ \vdots & \vdots \\ 1 & X_{iN} \end{bmatrix} \tag{18}$$

Let $\mathbf{Y}$ be the $N \times 1$ phenotype vector. Let $\mathbf{b} = (\mu, a)$ is the vector of effect parameters with priors $\mu \sim N(0, \sigma_u^2)$ and $a \sim N(0, \sigma_a^2)$. The Baye's factor is given by (supplementary material "Protocol S1: Analytical computations for prior D2" [4]),

$$BF(i) = \frac{|\Omega|^{1/2}}{\Omega_0^{1/2}\sigma_a} \left[\frac{\mathbf{Y}^t\mathbf{Y} - \mathbf{B}^t\Omega^{-1}\mathbf{B}}{\mathbf{Y}^t\mathbf{Y} - \Omega_0 N^2 \Omega^{-1}\overline{\mathbf{Y}}^2} \right] \tag{19}$$

where $\Omega = (\nu^{-1} + \mathbf{X}^t\mathbf{X})^{-1}$, $\Omega_0 = N^{-1}$, $\nu = diag(\sigma_u^2, \sigma_a^2)$ and $\mathbf{B} = \Omega\mathbf{X}^t\mathbf{Y}$. In the limit $\sigma_u \to \infty$, $\nu^{-1} = diag(0, \sigma_a^{-2})$. Also, let $\overline{X_i} = \sum_{j=1}^{N} X_{ij}$ and $\langle X_i, X_i \rangle = \sum_{j=1}^{N} X_{ij}^2$. Then,

$$\Omega^{-1} = \begin{bmatrix} N & N\overline{X_i} \\ N\overline{X_i} & N\langle X_i, X_i \rangle + \sigma_a^{-2} \end{bmatrix} \tag{20}$$

Therefore,

$$|\Omega^{-1}| = N^2[\langle X_i, X_i \rangle + N^{-1}\sigma_a^{-2} - \overline{X_i}^2] \tag{21}$$

and,

$$\Omega = \frac{1}{N^2[\langle X_i, X_i \rangle + N^{-1}\sigma_a^{-2} - \overline{X_i}^2]} \begin{bmatrix} N\langle X_i, X_i \rangle + \sigma_a^{-2} & N\overline{X_i} \\ N\overline{X_i} & N \end{bmatrix} = \Omega^t \tag{22}$$

The denominator of the term within brackets in Eqn.(19) is

$$\mathbf{Y}^t\mathbf{Y} - \Omega_0 N^2 \Omega^{-1}\overline{\mathbf{Y}}^2 = \mathbf{Y}^t\mathbf{Y} - N\overline{\mathbf{Y}}^2 = \langle \mathbf{Y} - \overline{\mathbf{Y}}, \mathbf{Y} - \overline{\mathbf{Y}} \rangle. \tag{23}$$

Now,

$$\begin{aligned}
\mathbf{B}^t\Omega^{-1}\mathbf{B} &= (\Omega\mathbf{X}^t\mathbf{Y})^t\Omega^{-1}(\Omega\mathbf{X}^t\mathbf{Y}) \\
&= (\mathbf{Y}^t\mathbf{X})\Omega^t(\mathbf{X}^t\mathbf{Y}) \\
&= N\begin{bmatrix}\overline{\mathbf{Y}} & \langle X_i, \mathbf{Y}\rangle\end{bmatrix}\Omega^t\begin{bmatrix}\overline{\mathbf{Y}} \\ \langle X_i, \mathbf{Y}\rangle\end{bmatrix} \qquad (24)
\end{aligned}$$

Combining Eqns 19, 22, 23 and 24, we see that only the genotype-genotype inner products $\langle X_i, X_i\rangle$, phenotype-phenotype inner product $\langle \mathbf{Y}-\overline{\mathbf{Y}}, \mathbf{Y}-\overline{\mathbf{Y}}\rangle$ and genotype-phenotype inner products $\langle \mathbf{Y}, X_i\rangle$ are sufficient to compute the Baye's factor for SNP i. As a result, *Bimbam* can be efficiently implemented for both individual level genotype data and summary data.

2.2 Computation of Bayes Factor using logistic regression

With binary logistic Regression, for a model containing a single SNP the genotype matrix $\mathbf{X}$ has N rows and 2 columns for the intercept and dosage. The phenotype vector is assumed to be binary containing values $\{1, -1\}$. The data log likelihood is

$$L(\mathbf{b}) = \log\Pr(\mathbf{Y}|\mathbf{b}, \mathbf{X}) = -\sum_{i=1}^{N}\log[1 + \exp(-Y_i {X_i}^T\mathbf{b})] \qquad (25)$$

where ${X_i}^T$ denotes the ith row (or sample) of $\mathbf{X}$. The prior distribution of the regression coefficients is assumed to be normal with mean 0 and covariance matrix ν, i.e, $\mathbf{b} \sim N(0, \nu)$. We assume $\nu = \mathrm{diag}(\sigma_u^2, \sigma_a^2)$, with prior $\Pr(\mathbf{b}) = 2\pi^{-1}\nu^{-0.5}\exp(-0.5\mathbf{b}^T\nu^{-1}\mathbf{b})$ for $\mathbf{b} = [b_u, b_a]^T$. Let H_{null} denotes the null hypothesis that no SNP is disease associated, and H_{alt} denotes the alternative event that SNP i is disease associated. The Bayes factor is

$$BF(i) = \frac{\int \Pr(Y|X, \mathbf{b}, H_{\text{alt}})\Pr(\mathbf{b}|H_{\text{alt}})d\mathbf{b}}{\int \Pr(Y|X, \mathbf{b}, H_{\text{null}})\Pr(\mathbf{b}|H_{\text{null}})d\mathbf{b}} \qquad (26)$$

The numerator can be evaluated as $\int \Pr(Y|X, \mathbf{b}, H_{\text{alt}})\Pr(\mathbf{b}|H_{\text{alt}})d\mathbf{b} = \int \Pr(Y|X, \mathbf{b})\Pr(\mathbf{b})d\mathbf{b} = \int f_{\text{alt}}(\mathbf{b})d\mathbf{b}$. The Laplace method approximates $f_{\text{alt}}(\mathbf{b})$ as $f_{\text{alt}}(\mathbf{b}) = f_{\text{alt}}(\hat{\mathbf{b}})\exp[-0.5(\mathbf{b}-\hat{\mathbf{b}})^T\Sigma_{\text{alt}}(\mathbf{b}-\hat{\mathbf{b}})] = f_{\text{alt}}(\hat{\mathbf{b}})(2\pi)|\Sigma_{\text{alt}}|^{-0.5}$, $\hat{\mathbf{b}}$ being the mode of the function $f_{\text{alt}}(\mathbf{b})$. The matrix Σ_{alt} is $-\nabla\nabla\log f_{\text{alt}}(\mathbf{b})|_{\mathbf{b}=\hat{\mathbf{b}}}$, $\hat{\mathbf{b}}$ being the mode of the function $f_{\text{alt}}(\mathbf{b})$ and Σ_{alt} is $-\nabla\nabla\log f_{\text{alt}}(\mathbf{b})|_{\mathbf{b}=\hat{\mathbf{b}}}$. Similarly, for the null model, the denominator is given by $\int f_{\text{null}}(\mathbf{b})d\mathbf{b} \approx f_{\text{null}}(\hat{\mathbf{b}})(2\pi)^{0.5}|\Sigma_{\text{null}}|^{-0.5}$.

To find the mode $\hat{\mathbf{b}}$, we employ the Fletcher-Reeves conjugate gradient algorithm implemented in the GNU Scientific Library (`http://www.gnu.org/software/gsl/manual/html_node/Multimin-Algorithms-with-Deriva` `html`). The maximization problem is converted to a minimization of objective function $F(\mathbf{b})$,

$$F_{\text{alt}}(\mathbf{b}) = -\log f(\mathbf{b}) = \sum_{i=1}^{N} \log[1 + \exp(-Y_i {X_i}^T \mathbf{b})] + \log(2\pi\sigma_u\sigma_a) + 0.5[(b_u/\sigma_u)^2 + (b_a/\sigma_a)^2]. \tag{27}$$

Then the hessian matrix Σ_{alt} is given by $X^T Q X + \nu^{-1}$ where Q is a $N \times N$ diagonal matrix with elements $q_{ii} = [1 + \exp(-Y_i {X_i}^T \mathbf{b})]^{-1}[1 + \exp(Y_i {X_i}^T \mathbf{b})]^{-1}$. The numerator of the Bayes Factor is evaluated as $\log(2\pi) - 0.5 \log |\Sigma_{\text{alt}}| - F_{\text{alt}}(\hat{\mathbf{b}})$.

For the null model (intercept only), $\mathbf{b} = b_u$, giving the objective function

$$F_{\text{null}}(\mathbf{b}) = -\log f(\mathbf{b}) = \sum_{i=1}^{N} \log[1 + \exp(-Y_i b_u)] + 0.5\log(2\pi) + \log(\sigma_u) + 0.5[(b_u/\sigma_u)^2] \tag{28}$$

The hessian is simply $\Sigma_{\text{null}} = \frac{N \exp b_u}{(1+\exp b_u)^2} + \sigma_u^{-2}$ and the denominator of the Bayes Factor is given by $0.5 \log(2\pi|\Sigma|^{-1}) - F_{\text{null}}(\hat{\mathbf{b}})$.

3 Simulations in the Absence of Genotype Data

Simulations replace strict genotype permutations when individual genotype data are not available. To evaluate the statistical significance, we use simulations to generate the null distribution of the test statistics. We improved the simulation method used in VEGAS [5] by using the LDL factorization instead of the Cholesky decomposition. The Cholesky decomposition requires the correlation matrix to be positive definite (PD). A correlation matrix is guaranteed PD only if the SNPs are linearly independent, which is not always satisfied in a densely genotyped genomic region. In addition, the correlation matrix that we work with may have defects that cause the matrices to be indefinite (see below, Sec. 4). Although the spectral decomposition method can restore a 'valid' correlation matrix from a defective one (Sec. 4), the resulting correlation matrix may still be positive semi-definite (PSD) due to the linearly dependent SNPs, or even indefinite due to round-off errors. Therefore, we used the LDL factorization which generalizes the Cholesky decomposition and can factorize any symmetric matrix.

Under the null, the z-score for a single SNP follows a t distribution with $N - 2$ degrees of freedom

(N is the number of individuals in the study). Because N is usually large in the meta-analysis, the t distribution approximates a normal distribution with mean 0 and variance 1. Assume $\mathbf{\Sigma}$ is the correlation matrix for the P SNPs in a gene. Under the null, the correlation matrix among the z-scores is also $\mathbf{\Sigma}$. Therefore, the null distribution of the z-scores for the P SNPs in a gene is multivariate normal with mean 0 and correlation matrix $\mathbf{\Sigma}$.

To simulate this distribution, we first sample a P-element vector Z with elements independently and identically distributed as $\text{Norm}(0, 1)$. The LDL factorization of the correlation matrix $\mathbf{\Sigma} = \mathbf{LDL}^T$, in which $\mathbf{L}$ is unit lower triangular and $\mathbf{D}$ is diagonal. If $\mathbf{\Sigma}$ is PSD (as in a 'valid' correlation matrix), the diagonals of $\mathbf{D}$ is non-negative (or positive if SNPs are linearly independent) and $\mathbf{D}^{1/2}$ exists. If the correlation matrix is defective and therefore not PSD, a spectral decomposition method (Sec. 4) constructs a PSD matrix that is 'similar' to the original correlation matrix. The constructed PSD matrix, instead of the original defective correlation matrix, is then LDL factorized. Due to round-off errors, diagonals of $\mathbf{D}$ from the factorization of the PSD matrix can be very small negative numbers instead of 0. If that happens, we set these elements of $\mathbf{D}$ to 0.

If Z is i.i.d. normal with mean 0 and variance 1, $\mathbf{LD}^{1/2}Z$ has multivariate normal distribution with mean 0 and variance $\mathbf{\Sigma}$. The simulated z-scores under the null are then $\mathbf{LD}^{1/2}Z$, which can be used to calculate the test statistics under the null and estimate the empirical P-value.

4 Creating a valid correlation matrix

GWiS relies on the LDL factorization of the SNP correlation matrix to perform simulations for the p-values. Ideally, a correlation matrix should be positive-semidefinite (PSD), which is a sufficient condition to guarantee solutions for the matrix factorization and to ensure that the diagonal matrix, $\mathbf{D}$, has non-negative elements. In practice, the correlation matrices may not be PSD. This can happen due to the missing genotype data, or due to the round-off error if the correlations are pre-calculated and saved to external files.

We use the spectral decomposition method to find a PSD matrix that is 'similar' to the original correlation matrix [6]. An alternative method proposed by Higham [7] is more rigorous and can estimate the 'nearest' PSD matrix. However, the Higham method is iterative and is computationally much more expensive. In practice, we find the spectral decomposition method performs well empirically and only

makes very small changes to the original correlation matrix.

For a correlation matrix $\mathbf{\Sigma}$, the spectral decomposition method first finds its eigenvalues, $\{\lambda_i\}$, and its eigensystem, $\mathbf{A}$, such that

$$\mathbf{\Sigma} \cdot \mathbf{A} = \mathbf{\Lambda} \cdot \mathbf{A}, \tag{29}$$

in which $\mathbf{\Lambda}$ is a diagonal matrix of the eigenvalues, $\{\lambda_i\}$. According to eigen decomposition, the real symmetric matrix $\mathbf{\Sigma}$ can be reconstructed from $\mathbf{\Lambda}$ and $\mathbf{A}$ as

$$\mathbf{\Sigma} = \mathbf{A}\mathbf{\Lambda}\mathbf{A}^t. \tag{30}$$

A naïve way to make $\mathbf{\Sigma}$ PSD is to set the negative eigenvalues in $\mathbf{\Lambda}$ to be 0,

$$\lambda'_i = \max(\lambda_i, 0). \tag{31}$$

The PSD matrix, $\mathbf{\Sigma}'$, is then reconstructed from $\{\lambda'_i\}$,

$$\mathbf{\Sigma}' = \mathbf{A}\mathbf{\Lambda}'\mathbf{A}^t, \tag{32}$$

in which $\mathbf{\Lambda}'$ is a diagonal matrix of the new eigenvalues, $\{\lambda'_i\}$. This method, although gives a PSD matrix, does not guarantee the unity of the diagonal elements, which is an essential property of the correlation matrix. A scaling matrix has to be introduced to scale the diagonals back to 1. This scaling matrix is diagonal and its elements are

$$t_i = \left(\mathbf{\Sigma}_j a_{ij}^2 \lambda'_j\right)^{-1}. \tag{33}$$

A correlation matrix corrected to be PSD with unit diagonals is

$$\begin{aligned} \mathbf{\Sigma}'' &= \sqrt{\mathbf{T}}\mathbf{\Sigma}'\sqrt{\mathbf{T}^t} \qquad (34) \\ &= \sqrt{\mathbf{T}}\mathbf{A}\mathbf{\Lambda}'\mathbf{A}^t\sqrt{\mathbf{T}^t}, \qquad (35) \end{aligned}$$

in which $\mathbf{T}$ is a diagonal matrix of $\{t_i\}$. The final correlation matrix σ'' is used for LDL factorization for simulations. The solution to the factorization is guaranteed and the elements of its solution matrix, $\mathbf{D}$, are non-negative.

References

1. Consortium IH (2005) A haplotype map of the human genome. Nature 437: 1299–1320.

2. Consortium GP, Durbin RM, Abecasis GR, Altshuler DL, Auton A, et al. (2010) A map of human genome variation from population-scale sequencing. Nature 467: 1061–1073.

3. Ortega JM, Rheinboldt WC (2000) Iterative Solution of Nonlinear Equations in Several Variables. Classics in Applied Mathematics. SIAM.

4. Servin B, Stephens M (2007) Imputation-based analysis of association studies: candidate regions and quantitative traits. PLoS Genet 3: e114.

5. Liu JZ, McRae AF, Nyholt DR, Medland SE, Wray NR, et al. (2010) A versatile gene-based test for genome-wide association studies. Am J Hum Genet 87: 139–145.

6. Rebonato R (1999) The most general methodology to create a valid correlation matrix for risk management and option pricing purposes. Quantitative Research Centre of the NatWest ... .

7. Higham N (2002) Computing the nearest correlation matrix—a problem from finance. IMA Journal of Numerical Analysis 22: 329.
