# Supplementary material for: Fast Association Tests for Genes with FAST
Source: PLoS One. 2013 Jul 23;8(7):e68585. doi: 10.1371/journal.pone.0068585 (PMC3720833; doi:10.1371/journal.pone.0068585)
Supplement: Materials S2 — Figure S1, Estimated power of the methods to detect the simulated gene under linear and logistic regression models. Figure S2, Comparing single SNP chi-squares and p-values between linear and logistic regression with genotype data for N = 1000, 3000 and 5000. Figure S3, Comparing minSNP Gene test statistic and gene p-values between logistic regression (genotype data) and linear regression (summary data) and for N = 1000, 3000 and 5000. Figure S4, Comparing Vegas test statistic and gene p-values between logistic regression (genotype data) and linear regression (summary data) for N = 1000, 3000 and 5000. Figure S5, Comparing Bimbam test statistic and gene p-values between logistic regression (genotype data) and linear regression (summary data) for N = 1000, 3000 and 5000. Figure S6, Comparing GWiS test statistic and gene p-values between logistic regression (genotype data) and linear regression (summary data) for N = 1000, 3000 and 5000. Only models with test statistic >0 undergo permutations to generate P-values. Figure S7, Comparing Gates p-values between logistic regression (genotype data) and linear regression (summary data) for N = 1000, 3000 and 5000. Figure S8, Comparing minSNP test statistic and gene p-values between logistic regression (genotype data) and linear regression (summary data) for N = 1000, 3000 and 5000. (PDF) [file pone.0068585.s002.pdf]

FAST : Supplementary 2

Pritam Chanda, Hailiang Huang, Dan E. Arking, Joel S. Bader

Additional Experiments

In absence of genotype data, when the phenotype is case-control, FAST uses linear regression to approximate the calculations of the test statistics for the two Bayesian methods GWiS and BIMBAM. This is necessary because the computation of the test statistics using logistic regression is not possible with just summary data (single SNP regression coefficients and standard errors) as maximum log-likelihood estimations in logistic regression do not have closed form solutions and need numerical optimization algorithms such as gradient descent to be estimated. We show below that for SNPs with small effect sizes and higher significance, test statistics and the associated p-values computed with summary data (using linear regression model) data are highly concordant with those computed using logistic regression using genotype data.

Data with case:control ratio = 1:4 and 50 SNPs were simulated using Plink for total sample sizes of 1000, 3000 and 5000 individuals. Out of the 50 SNPs, 5 SNPs were simulated to be disease associated with a multiplicative risk of 1.2 for the homozygotes. All SNPs are mapped to a single gene. First, each test statistic was computed under logistic regression model with genotype data and p-values were obtained with 10000 permutations. Next, using summary data from logistic regression, FAST re-computed each test statistic and p-values were obtained with 10000 permutations (simulation procedure described in the main text for summary data). Figure 1 shows the power to detect the gene as significant out of 100 independent repetitions of the above simulation procedure across each method and different sample sizes. Figure 2-8 shows the extremely high concordance between the test statistics and corresponding p-values for each method. The concordance is higher for SNPs and genes that have smaller p-values (i.e. those that are more likely to be disease associated).

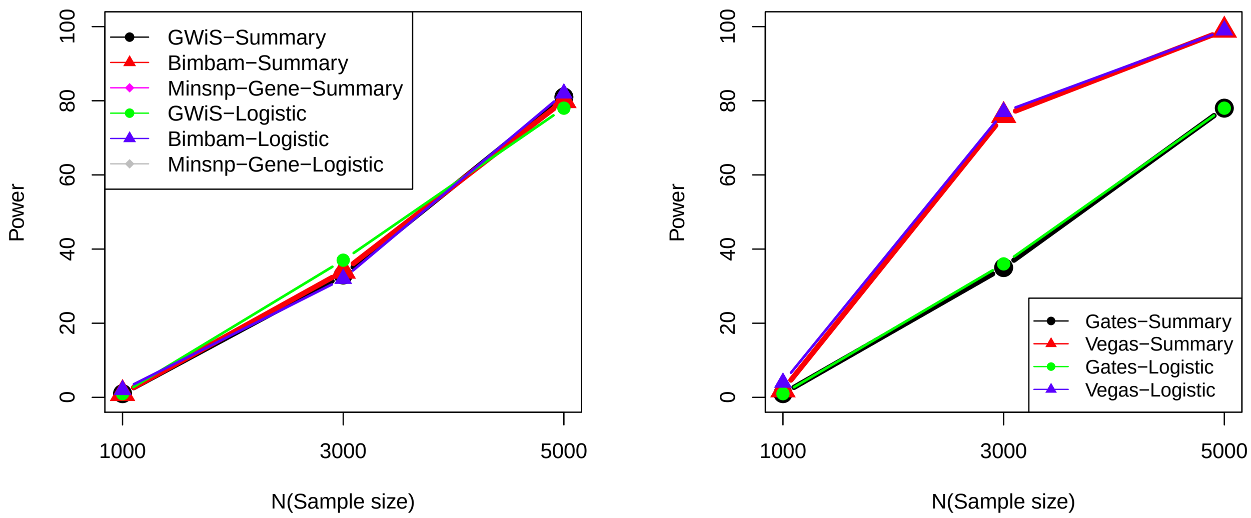

Figure S1: Estimated power of the methods to detect the simulated gene under linear and logistic regression models.

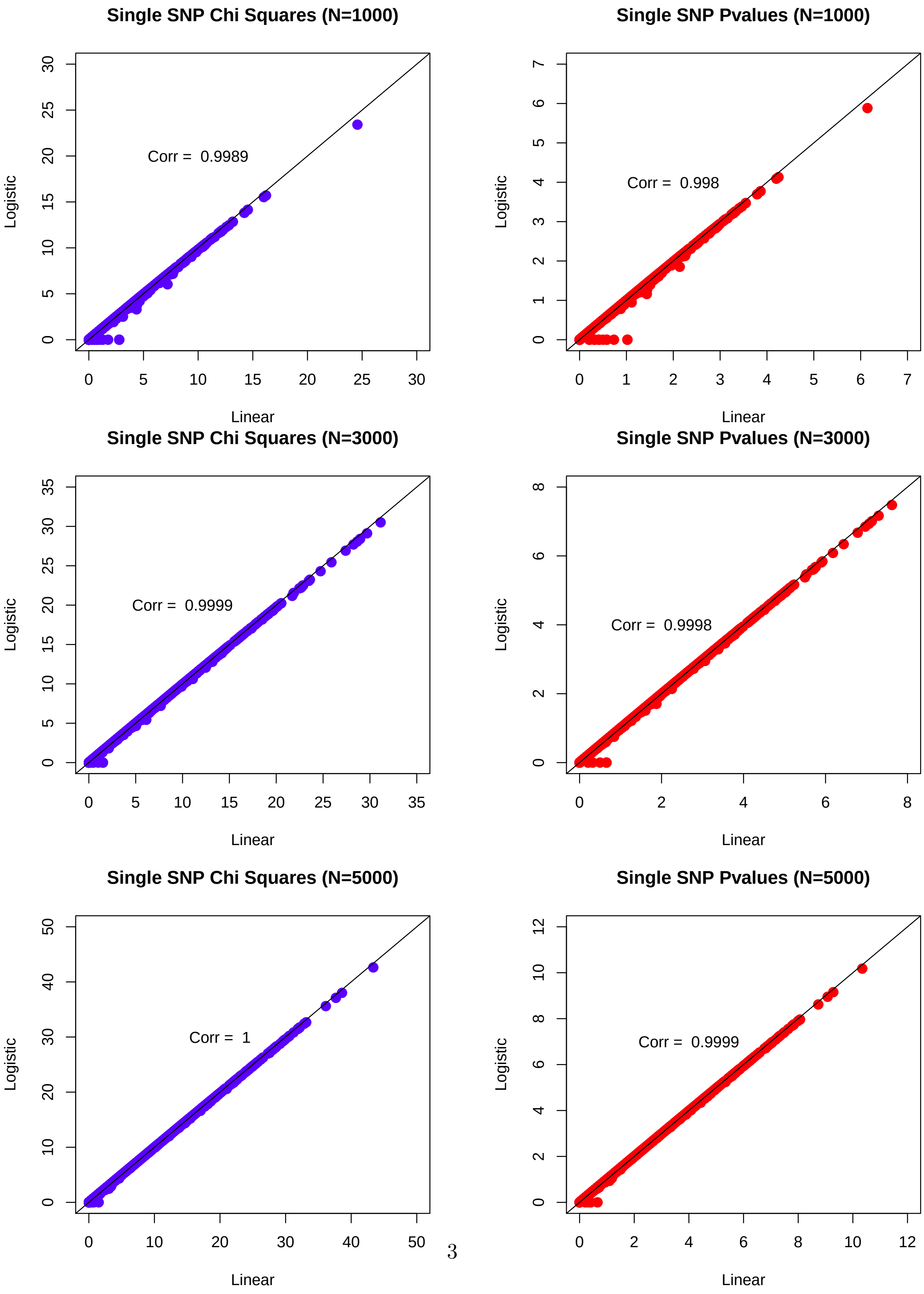

Figure S2: Comparing single SNP chi-squares and p-values between linear and logistic regression with genotype data for N=1000, 3000 and 5000

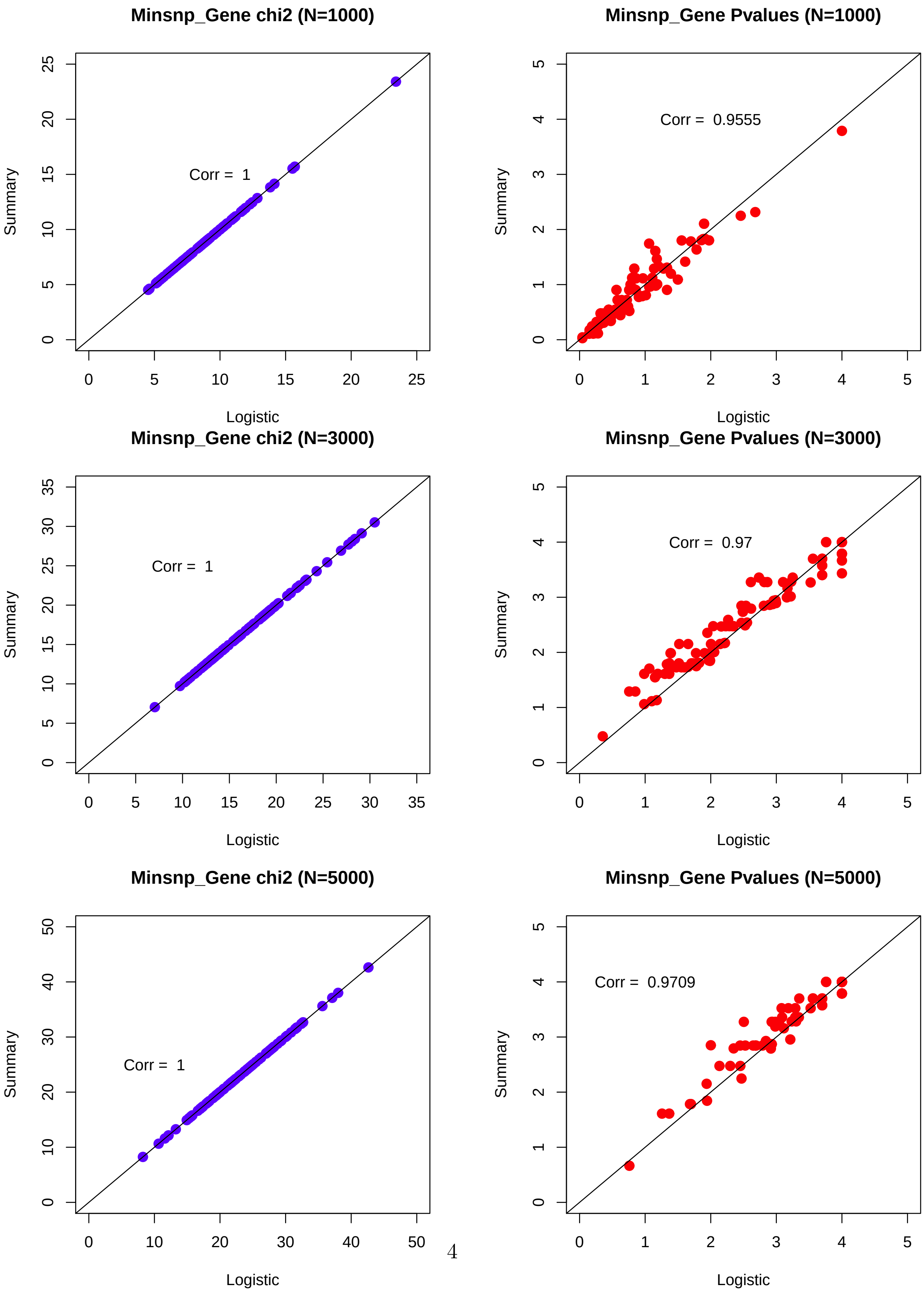

Figure S3: Comparing minSNP_Gene test statistic and gene p-values between logistic regression (genotype data) and linear regression (summary data) and for N=1000, 3000 and 5000

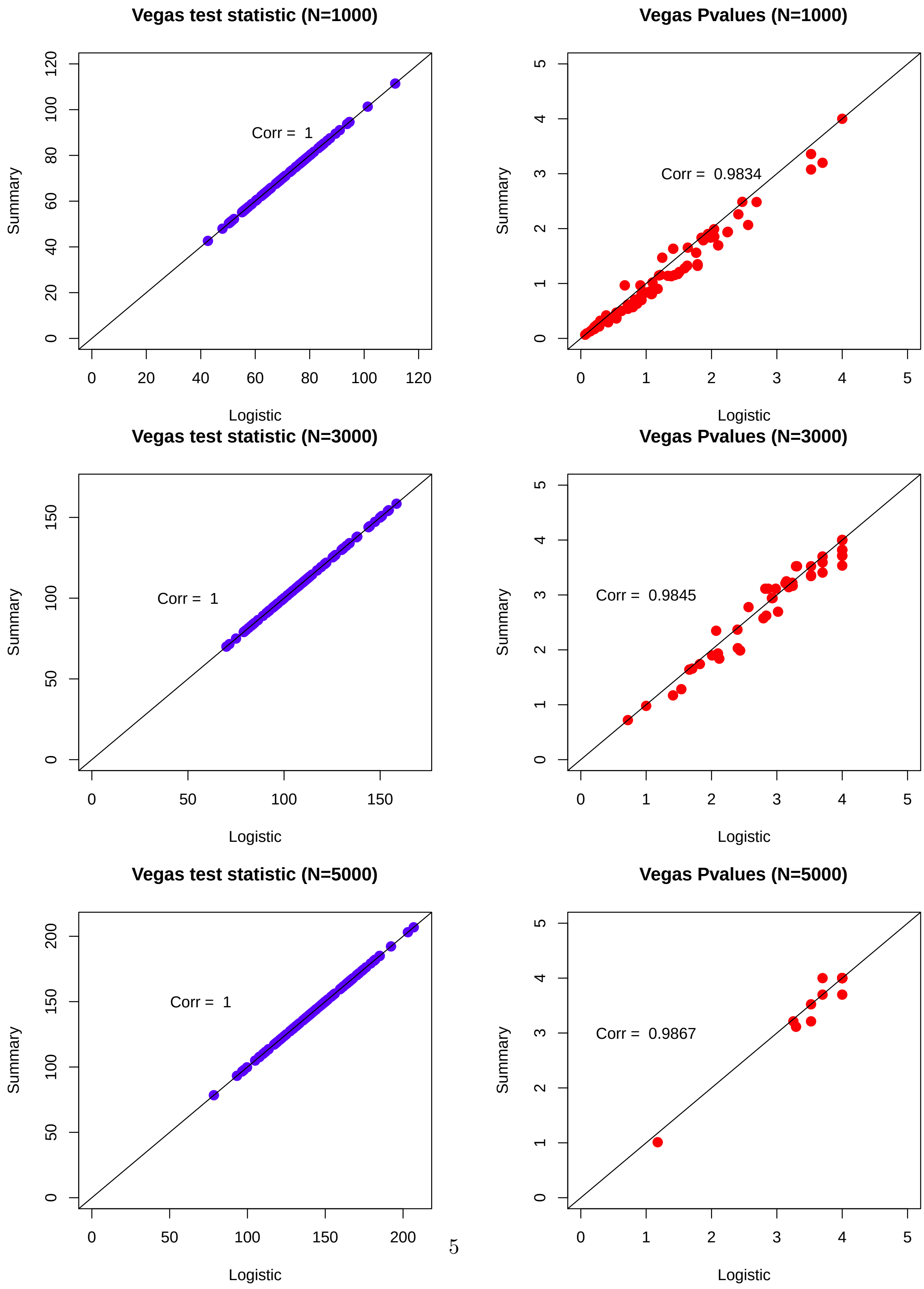

Figure S4: Comparing Vegas test statistic and gene p-values between logistic regression (genotype data) and linear regression (summary data) for N=1000, 3000 and 5000

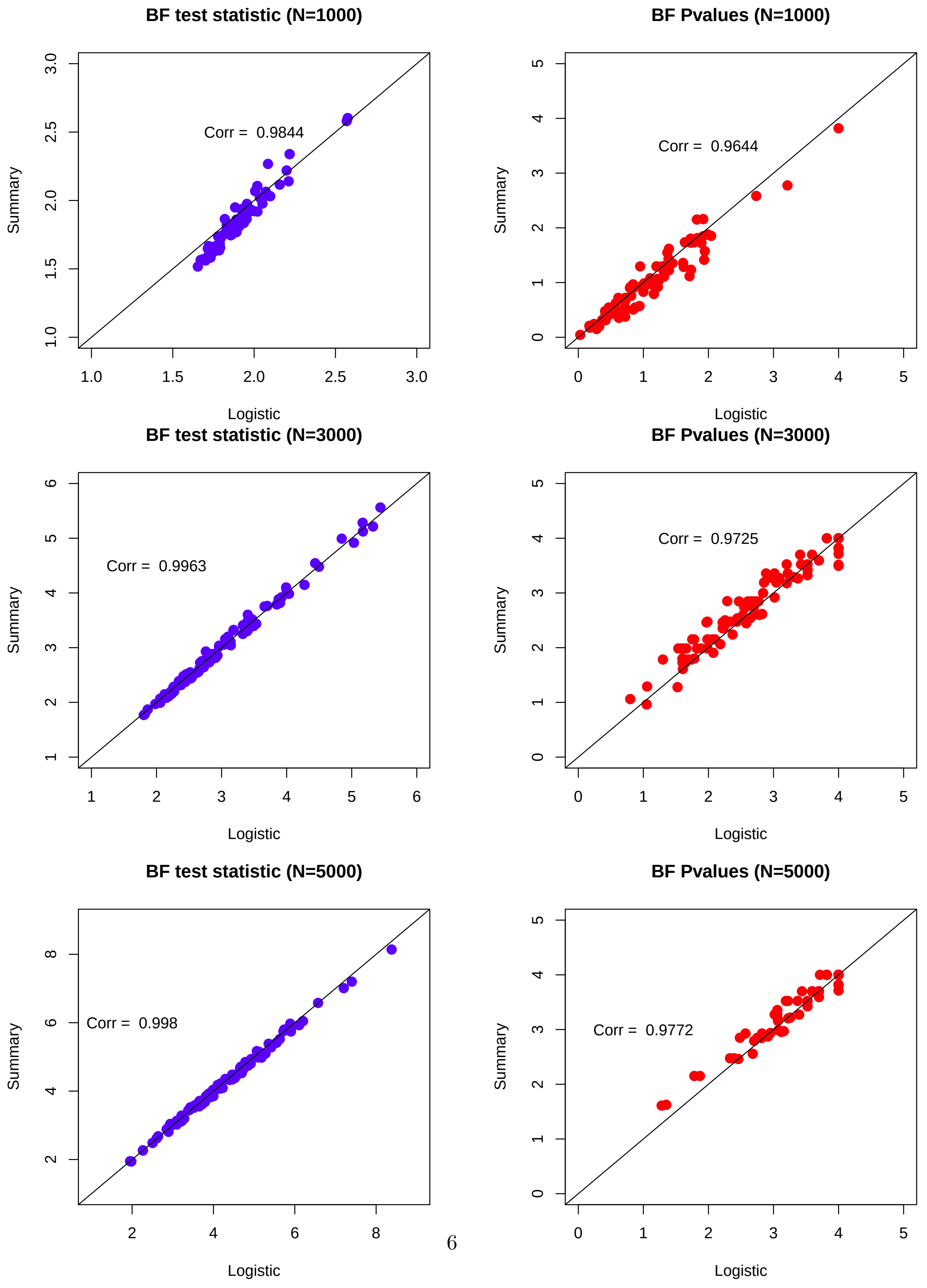

Figure S5: Comparing Bimbam test statistic and gene p-values between logistic regression (genotype data) and linear regression (summary data) for N=1000, 3000 and 5000

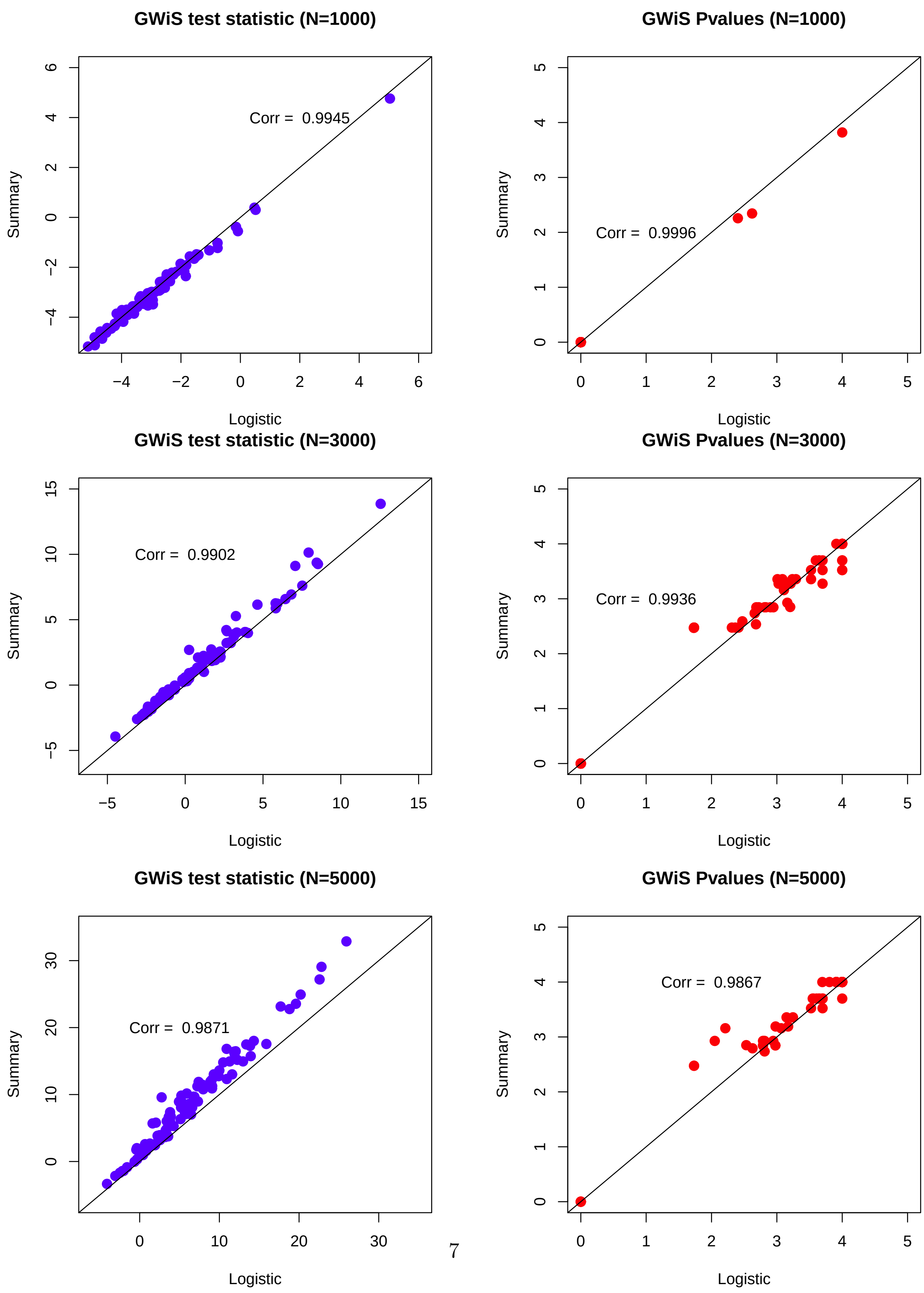

Figure S6: Comparing GWiS test statistic and gene p-values between logistic regression (genotype data) and linear regression (summary data) for N=1000, 3000 and 5000. Only models with test statistic > 0 undergo permutations to generate P-values.

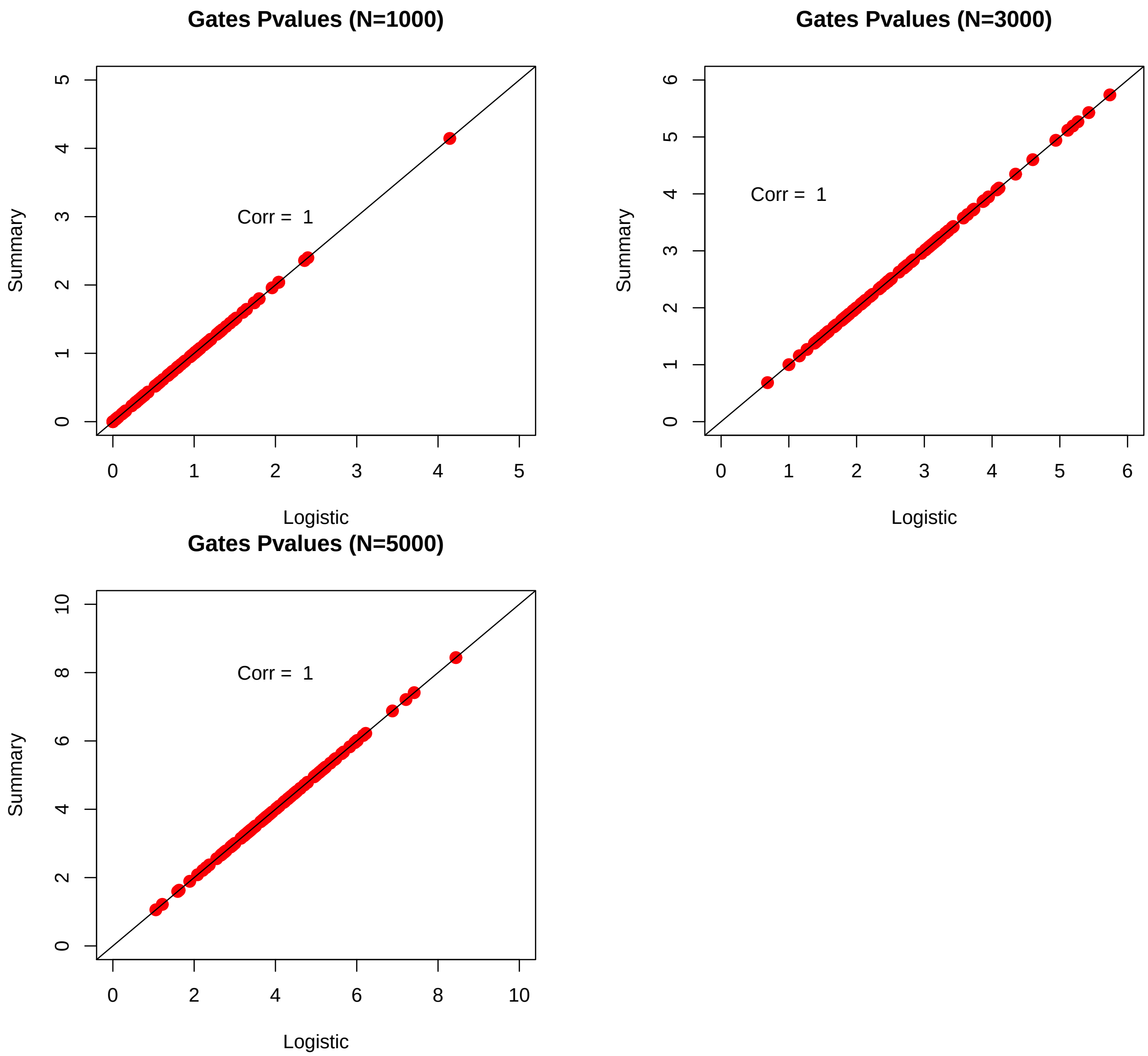

Figure S7: Comparing Gates p-values between logistic regression (genotype data) and linear regression (summary data) for N=1000, 3000 and 5000

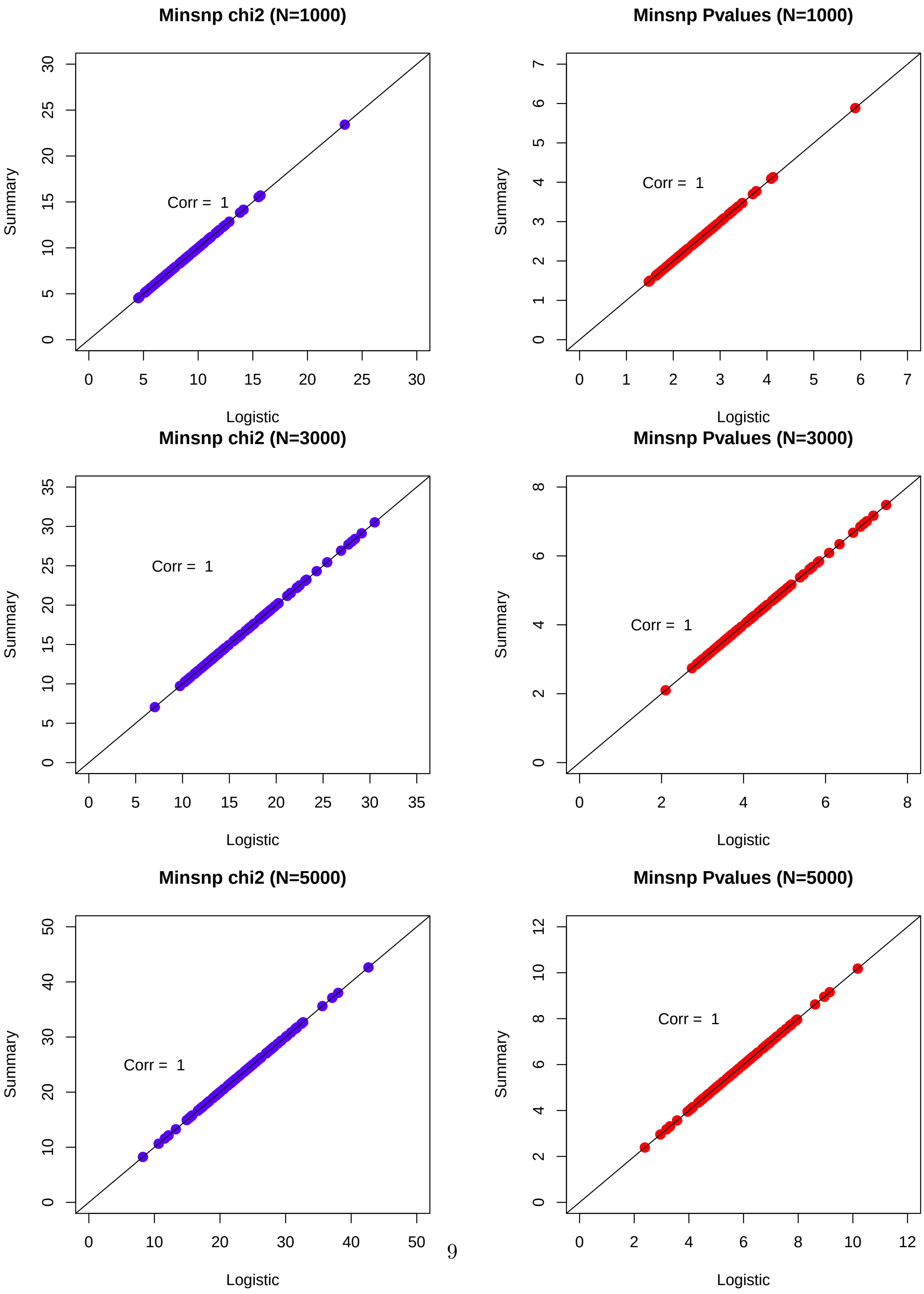

Figure S8: Comparing minSNP test statistic and gene p-values between logistic regression (genotype data) and linear regression (summary data) for N=1000, 3000 and 5000
